# Factors associated with the use of dental services by older people in the city of São Paulo, Brazil

**DOI:** 10.1590/1980-549720250042.2

**Published:** 2025-08-04

**Authors:** Yan Nogueira Leite de Freitas, Cristian Arnecke Schröder, Camila Nascimento Monteiro, Edigê Felipe de Sousa Santos, Moisés Goldbaum, Chester Luiz Galvão Cesar, Marília Cristina Prado Louvison

**Affiliations:** I Universidade Federal do Amazonas, School of Dentistry - Manaus (AM), Brazil.; II Universidade de São Paulo, School of Public Health - São Paulo (SP), Brazil.; III Hospital Sírio-Libanês - São Paulo (SP), Brazil.; IV Universidade de São Paulo, School of Medicine, Department of Preventive Medicine- São Paulo (SP), Brazil.

**Keywords:** Aged, Equity in access to health services, Health surveys, Dental health services

## Abstract

**Objective:**

The aim of this study was to investigate the factors associated with the use of dental services by older people living in São Paulo, Brazil.

**Methods:**

A cross-sectional study was conducted using secondary data analysis from sub-samples of older people in the Health Surveys in the City of São Paulo, Brazil (ISA-Capital), conducted in 2003, 2008 and 2015. Several variables were tested as factors associated with the outcome “dental visit in the last year”. Adjusted odds ratios were estimated from three binary logistic regression models, one for each year.

**Results:**

Sex was associated with the outcome only in 2008, with an adjusted odds ratio (ORa) of 0.65 (0.48-0.89) for females. Younger individuals (up to 74 years old) were more likely to have seen a dentist in 2008 and 2015. The variable years of study remained associated with the outcome throughout the entire period, revealing an ORa of 8.22 (4.23-15.99) for individuals with 12 or more years of study in 2003. This measure decreased to 1.77 (1.25-2.51) in 2015. Race remained an associated factor only in 2003 [ORa=1.50 (1.04-2.17)] in favor of self-declared white individuals. On the other hand, monthly income and health insurance were factors associated with the outcome only in 2008 and 2015.

**Conclusion:**

The findings of this study point to critical issues that deserve the attention of managers, on the basis of intersectoral measures that contribute to active and healthy aging.

## INTRODUCTION

Several studies have shown that inequalities in the use of health services represent a global challenge, even in countries that have universal health systems, such as Brazil^1^ ^,^ ^2^ ^,^ ^3^ ^,^ ^4^ ^,^ ^5^ ^,^ ^6^. With regard to oral health services, these inequalities are even more striking, since dentistry was for years on the fringes of public health policies^7^ ^,^ ^8^. It is known that oral health care for the Brazilian population, for example, was born from a curative and mutilating logic - focusing on diseases and injuries in children and adolescents -, reflecting a model of exclusion from access to dental services for the elderly, with little impact on epidemiological indicators^9^ ^,^ ^10^.

Since 2004, the Brazilian Unified Health System (SUS) has been outlining guidelines to overcome inequalities in the use of and access to dental services, which culminated in the National Oral Health Policy (PNSB), also known as “Smiling Brazil”. The main objective of this policy is to reduce inequalities in oral health care, expanding the population’s access to comprehensive dental services and focusing on primary health care (PHC)^11^.

Since the implementation of the PNSB, there has been an increase in the number of oral health professionals working in the SUS, especially with the incentives for Oral Health Teams (eSB) in PHC. Thus, it can be observed, for example, that in 2024 there were 35,827 eSB linked to the SUS, guaranteeing 50.65% coverage of the Brazilian population. In the city of São Paulo, for the same year, in April, there were 640 eSB contracted, which guaranteed only 18.56% coverage, according to data made available by the e-Gestor AB platform of the Ministry of Health (MS) (https://egestorab.saude.gov.br/). It is therefore observed that despite the incentives aimed at improving access to dental services by the MS in recent years, coverage by PHC is still low in the city of São Paulo^10^.

In this context, the use of dental services has been the subject of research in recent decades. It has been observed that elderly individuals with lower income and education levels are those who have the greatest difficulty in seeiing a dentist receiving regular follow-up from a dental surgeon-dentist, ^1^ ^,^ ^10^ ^,^ ^12^ ^,^ ^13^ ^,^ ^14^.

In the case of Brazil, its territorial size and regional heterogeneities contribute to the low coverage of dental services offered by the SUS, especially specialized services, which are in high demand among the older population. In this context, these inequalities can be perceived even more easily, since a large part of the demand is directed to supplementary health care or private consultations for direct payment, reinforcing the disparities in access to oral health in this population stratum. Therefore, it is understood that these inequalities are better explained by socioeconomic and demographic determinants than by the oral health of these individuals^13^.

Among the different realities in Brazil, the city of São Paulo stands out for having the highest absolute number of older people in the country^15^. Regarding inequalities in the use of dental services in this municipality, Monteiro et al.^16^ observed an increase in the use of oral health services by the adult population between 2003 and 2008. However, this increase was associated with individuals who were white and had higher levels of education, higher income, better housing conditions and health insurance. The authors also point out that there was a small increase in the use of these services in the public sector by the population with lower socioeconomic status. Therefore, determining whether these and other determinants are also related to the use of these services by an age group that lacks oral health care^1^ ^,^ ^12^ ^,^ ^13^, in the context of the city of São Paulo, can contribute to the discussion of measures and/or policies to improve this scenario.

In view of this scenario, this study aimed to investigate the factors associated with the use of dental services by older people in the city of São Paulo, Brazil, in the years 2003, 2008 and 2015.

## METHODS

To meet the proposed objective, a serial cross-sectional study was conducted with analysis of secondary data from subsamples of older people from the Health Surveys in the Municipality of São Paulo/Brazil (ISA-Capital), carried out in 2003, 2008 and 2015.

According to the Brazilian Institute of Geography and Statistics (IBGE), in 2022 the population of the municipality of São Paulo was 11,451,999 inhabitants, of which 2,027,003 were older people, aged 60 or over, representing 17.7% of the resident population^15^.

ISA-Capital is a population-based cross-sectional study carried out in the municipality of São Paulo, through household interviews. In 2003, 3,357 individuals were interviewed, of whom 872 (25.97%) were aged 60 or over, based on a two-stage cluster stratified sampling (census tracts and households)^17^. Following this methodology, in 2008 a stratified sampling by the same clusters selected 3,271 individuals, of whom 924 (28.25%) were elderly people^18^. In 2015, the sampling maintained the two-stage cluster logic, but for the first time the census tracts were grouped according to the five regional health coordination offices of the municipality: North, South, East, Central-West and Southeast. Thus, 3,184 individuals were selected to compose the sample, of which the older age group corresponded to 32% of them, totaling 1,019 individuals^19^. Further methodological details of these surveys can be found on the ISA-Capital website (https://www.fsp.usp.br/isa-capital/metodologia/).

For data tabulation, questionnaires administered in 2003, 2008, and 2015 were considered, organized into thematic blocks composed mostly of multiple-choice questions. Data were collected by trained interviewers and the questions were answered directly by residents of the selected households.

The outcome variable was the question regarding dental visits in the last year, categorized as yes/no, which was taken from block G of the questionnaires, which concerns the use of health services. The independent variables were selected according to Andersen’s Behavioral Model^20^ and also according to the literature, because they are recognized as factors associated with the use of dental services^21^. Therefore, the following were considered independent: sex (male/female); age (up to 74 years/75 years or older); color/race (white/black/brown/yellow/indigenous); marital status (married/unmarried); years of study (none or up to 3 years/4 to 7 years/8 to 11 years/12 or more years); monthly income (less than 1 minimum wage (MW)/between 1 and 2.5 MW/more than 2.5 to 6 MW/more than 6 MW); health problem in the last 15 days (yes/no) and multimorbidity (yes/no), considered as two or more self-reported chronic diseases.

It is worth noting that some independent variables were not considered for the three surveys, as there were changes over the years in the questionnaires. Thus, self-perception of general health (positive/negative) was considered for the 2003 and 2015 surveys; on the other hand, self-perception of oral health was only considered for the 2015 survey. Having health insurance (yes/no) was only considered for the 2008 and 2015 surveys. Polypharmacy (yes/no), considered as the concomitant use of four or more medications daily, was selected for the 2003 and 2015 surveys. The frequency of tooth brushing (never/does not brush every day/once a day/2 or more times a day) and frequency of changing the toothbrush (less than 3 months/between 3 and less than 6 months/between 6 months and less than 1 year/more than 1 year/never) were variables observed only in the 2015 survey.

Initially, a descriptive analysis was conducted for each of the three subsamples investigated, from which the prevalence of the outcome was observed among the categories of the independent variables, whose significance was tested based on Pearson’s ꭓ^2^ test, for a 95% confidence level. Next, the crude and adjusted effect measures (odds ratio-OR) were tested, considering the same confidence level. The adjusted measures were estimated from a binary logistic regression, maintaining the confidence level. To compose the multiple analysis, the variables with p≤0.25 in the crude analysis were selected.

### Data availability statement

The dataset supporting the findings of this study is not publicly available.

## RESULTS

The use of dental services was distributed heterogeneously across the three surveys. The variables color/race, years of education, and income were associated with the outcome in the three years studied (with p-values ranging from ≤0.001 to 0.01), where the prevalence of dental visits in the last year was higher among individuals who self-declared as white and yellow, with 12 or more years of education, and who earned more than 6 MW per month. Having health insurance was also associated with the outcome in 2008 and 2015 (p≤0.001), where individuals who reported having health insurance visited the dentist more often, as shown in Table 1.

**Table 1.** Descriptive analysis of data related to ISA-Capital 2003, 2008 and 2015. São Paulo, Brazil, 2025.

Variable	Dental visit last year (2003) n=872			Dental visit last year (2008) n=924			Dental visit last year (2015) n=1,019		
	Yes (%)	No (%)	p-value*	Yes (%)	No (%)	p-value*	Yes (%)	No (%)	p-value*
Sex									
Male	122 (29.8)	288 (70.2)	0.381	128 (36.0)	228 (64.0)	0.153	147 (38.0)	240 (62.0)	0.066
Female	119 (27.0)	321(73.0)		231 (40.7)	337 (59.3)		277 (43.8)	355 (56.2)	
Age (years)									
Up to 74	189 (29.6)	450 (70.4)	0.168	259 (42.1)	356 (57.9)	**0.004**	339 (44.7)	419 (55.3)	**0.001**
75 or older	52 (24.6)	159 (75.4)		100 (32.4)	209 (67.6)		85 (32.6)	176 (67.4)	
Skin color/race									
While	188 (31.9)	401 (68.1)	**0.01**	277 (42.3)	378 (57.7)	**0.0002**	269 (45.1)	328 (54.9)	**0.001**
Black	11(18.6)	48(81.4)		14 (26.4)	39 (73.6)		24 (30.4)	55 (69.6)	
Brown	35 (20.1)	139 (79.9)		53 (28.6)	132 (71.4)		77 (34.2)	148 (65.8)	
Yellow	7 (28.0)	18 (72.0)		13 (46.4)	15 (53.6)		21 (61.8)	13 (38.2)	
Schooling (years)									
None/up to 3	64 (17.9)	294 (82.1)	**≤0.001**	79 (25.2)	235 (74.8)	**≤0.001**	49 (25.0)	147 (75.0)	**≤0.001**
4 to 7	88 (28.6)	220 (71.4)		124 (35.5)	225 (64.5)		120 (37.6)	199 (62.4)	
8 to11	44 (38.9)	69 (61.1)		103 (54.8)	85 (45.2)		111 (43.0)	147 (57.0)	
12 or more	42 (73.7)	15 (26.3)		52 (73.2)	19 (26.8)		144 (58.5)	102 (41.5)	
Monthly income^†^ (minimum wage)									
Less than 1	25 (25.0)	75 (75.0)	**≤0.001**	13 (22.4)	45 (77.6)	**≤0.001**	41 (31.8)	88 (68.2)	**≤0.001**
Between 1 and 2.5	44 (18.3)	196 (81.7)		105 (31.0)	234 (69.0)		97 (33.0)	197 (67.0)	
More than 2.5 to 6	91 (31.1)	202 (68.9)		80 (44.2)	101 (55.8)		108 (42.4)	147 (57.6)	
More than 6	81 (37.3)	136 (62.7)		161 (46.5)	185 (53.5)		178 (52.2)	163 (47.8)	
Health insurance									
Yes	-	-		223 (50.3)	220 (49.7)	**≤0.001**	306 (48.8)	321 (51.2)	**≤0.001**
No	-	-		136 (28.3)	345 (71.7)		118 (30.3)	271 (69.7)	

*Significant Pearson’s X^2^ test; ^†^minimum wage was R$ 240 in 2003, R$ 415 in 2008 and R$ 788 in 2015.

Values in bold indicate significant associations.

As for the effect measures, in general, high OR was observed that decreased over time. The variable years of study exemplifies these findings, since individuals who in 2003 had studied for 12 or more years were approximately 8.36 (4.54-15.40) times more likely to have visited a dentist in the last year, compared to those who had no schooling or studied for only three years. When adjusted for the other variables, this statistic drops to 8.22 (4.23-15.99). However, in 2015, a decrease was observed in these measures, with a crude OR of 2.49 (1.85-3.33) and an adjusted OR of 1.77 (1.25-2.51), indicating a possible reduction in inequalities in the use of oral health services in relation to education in the subsamples studied, as shown in Tables 2 and 3.

**Table 2.** Crude odds ratios of ISA-Capital 2003, 2008 and 2015. São Paulo, Brazil, 2025.

Variable	Dental visit last year (2003) n=872			Dental visit last year (2008) n=924			Dental visit last year (2015) n=1,019		
	OR	(95%CI)	p-value*	OR	(95%CI)	p-value*	OR	(95%CI)	p-value*
Sex									
Male	1.14	(0.85-1.54)	0.381	0.82	(0.62-1.08)	0.153	0.78	(0.61-1.02)	0.066
Female	1			1			1		
Age (years)									
Up to 74	1.28	(0.90-1.83)	0.168	**1.52**	**(1.14-202)**	0.004	**1.67**	**(1.25-2.25)**	0.001
75 or older	1			1			1		
Skin color/race									
White	**1.84**	**(1.30-2.60)**	0.001	**1.67**	**(1.23-2.26)**	0.001	**1.41**	**(1.09-1.82)**	0.008
Brown/Black	1			1			1		
Married status									
Married	1.21	(0.87-1.67)	0.229	1.00	(0.77-1.31)	0.957	1.02	(0.79-1.30)	0.894
Unmarried	1			1			1		
Schooling (years)									
None/up to 3	1			1			1		
4 to 7	1.02	(0.75-1.39)	0.915	0.79	(0.61-1.05)	0.106	0.79	(0.60-1.03)	0.081
8 to 11	**1.75**	**(1.16-2.64)**	0.007	**2.27**	**(1.64-3.14)**	≤0.001	1.08	(0.81-1.44)	0.594
12 or more	**8.36**	**(4.54-15.40)**	≤0.001	**4.87**	**(2.83-8.38)**	≤0.001	**2.49**	**(1.85-3.33)**	≤0.001
Monthly income^†^ (minimum salary)									
Less than 1	1			1			1		
Between 1 and 2.5	**0.42**	**(0.33-0.68)**	≤0.001	**0.58**	**(0.44-0.78)**	≤ 0.001	**0.60**	**(0.45-0.80)**	≤0.001
More than 2.5 to 6	1.22	(0.90-1.67)	0.204	1.32	(0.95-1.83)	0.1	1.04	(0.78-1.39)	0.781
More than 6	**1.76**	**(1.27-2.44)**	0.001	**1.67**	**(1.27-2.19)**	≤ 0.001	**1.92**	**(1.47-2.50)**	≤0.001
Health problems in the last 15 days									
Yes	0.84	(0.60-1.19)	0.332	0.91	(0.67-1.24)	0.559	1.03	(0.77-1.38)	0.853
No	1			1			1		
General health self-perception									
Positive	1.33	(0.86-2.03)	0.196	-	-		**1.38**	**(1.07-1.78)**	0.014
Negative	1			-	-		1		
Multimorbidity									
Yes	0.99	(0.74-1.34)	0.992	0.84	(0.64-1.11)	0.222	0.89	(0.69-1.15)	0.380
No	1			1			1		
Polypharmacy									
Yes	0.84	(0.57-1.24)	0.373	-	-		1.15	(0.89-1.48)	0.278
No	1			-	-	1			
Health insurance									
Yes	-	-		**2.57**	**(1.96-3.38)**	≤ 0.001	**2.19**	**(1.68-2.86)**	≤0.001
No	-	-		1			1		
Oral health self-perception									
Positive	-	-		-	-		**1.33**	**(1.03-1.73)**	0.03
Negative	-	-		-	-		1		
Tooth brushing frequency									
Never	-	-		-	-		1		
Once a day	-	-		-	-		**0.32**	**(0.21-0.51)**	≤0.001
Twice or more often a day	-	-		-	-		**3.37**	**(2.22-5.11)**	≤0.001
Tooth brush replacement (months)									
More than 3	-	-		-	-		1		
Between 3 and 6	-	-		-	-		1.27	(0.98-1.63)	0.07
6 or more	-	-		-	-		**0.57**	**(0.42-0.78)**	≤0.001

OR: crude odds ratio.

*significant odds ratio; ^†^minimum wage in 2003 was R$ 240, R$ 415 in 2008 and R$ 788 in 2015.

Values in bold indicate significant odds ratio according to confidence interval.

**Table 3.** Adjusted odds ratio of ISA-Capital 2003, 2008 and 2015. São Paulo, Brazil, 2025.

Variable	Dental visit last year (2003) n=872			Dental visit last year (2008) n=924			Dental visit last year (2015) n=1,019		
	OR	(95%CI)	p-value*	OR	(95%CI)	p-value*	OR	(95%CI)	p-value*
Sex									
Male	-	-		**0.65**	**0.48-0.89)**	0.008	0.813	(0.61-1.08)	0.148
Female	-	-		1	-		1	-	
Age (years)									
Up to 74	1.13	(0.78-1.65)	0.51	**1.50**	**(1.09-2.04)**	0.012	**1.60**	**(1.17-2.20)**	0.003
75 or older	1	-		1	-		1		
Skin color/race									
White	**1.50**	**(1.04-2.17)**	0.032	1.16	(0.83-1.61)	0.38	1.23	(0.93-1.61)	0.143
Brown/Black	1	-		1	-		1	-	
Marital status									
Married	0.99	(0.70-1.39)	0.935						
Unmarried	1	-							
Schooling (years)									
None/up to 3	1	-		1	-		1		
4 to 7	-	-		**1.53**	**(1.08-2.16)**	0.018	1.12	(0.82-1.53)	0.487
8 to 11	**1.94**	**(1.25-3.00)**	0.003	**2.83**	**(1.86-4.31)**	≤0.001	-	-	
12 or more	**8.22**	**(4.23-15.99)**	≤0.001	**5.93**	**(3.15-11.17)**	≤0.001	**1.77**	**(1.25-2.51)**	0.001
Monthly income^†^ (minimum wage)									
Less than 1	1	-		1	-		1		
Between 1 and 2.5	0.79	0.45-1.41)	0.433	1.64	(0.82-3.30)	0.163	0.99	(0.71-1.40)	0.982
More than 2.5 to 6	1.41	(0.82-2.41)	0.213	1.95	(0.93-4.01)	0.077	-	-	
More than 6	1.11	(0.62-1.98)	0.724	**2.05**	**(1.02-4.10)**	0.043	**1.62**	**(1.18-2.23)**	0.003
General health self-perception									
Positive	1.00	(0.64-1.57)	0.99				1.11	(0.84-1.48)	0.465
Negative	1	-					1	-	
Multimorbidity									
Yes	-	-		1.02	(0.75-1.39)	0.895			
No	-	-		1	-				
Health insurance									
Yes	-	-		**1.98**	**(1.47-2.67)**	≤ 0.001	**1.69**	**(1.26-2.26)**	≤0.001
No	-	-		1	-		1	-	
Oral health self-perception									
Positive							1.14	(0.86-1.52)	0.351
Negative							1	-	
Tooth brushing frequency									
Never	-						1	-	
Once a day	-	-		-	-		1.11	(0.34-3.62)	0.858
Twice or more often a day	-	-		-	-		2.51	(0.824-7.63)	0.105
Tooth brush replacement (months)									
Less than 3	-						1	-	
Between 3 and 6	-	-		-	-		1.12	(0.83-1.51)	0.471
6 or more	-						0.71	(0.48-1.04)	0.076

*significant odds ratio; ^†^minimum wage in 2003 was R$ 240, R$ 415 in 2008 and R$ 788 in 2015.

Values in bold indicate significant odds ratio according to confidence interval.

Table 3 presents the best-fit model in the multiple analysis for each of the surveys studied. Thus, it is observed, for example, that in 2003, individuals who declared themselves white were more likely to have visited a dentist in the last year, when compared to brown or black individuals [OR=1.50 (1.04-2.17)]. Regarding sex, in 2008, male individuals were less likely to have visited a dentist in the last year [OR=0.65 (0.48-0.89)]. Income remained associated with the outcome in the last two surveys, revealing that in 2008, individuals who earned more than 6 MW were approximately 2.05 (1.09-2.11) times more likely to have visited a dentist in the last year when compared to the lowest income bracket. In 2015, a slight decrease in this possible income inequality was observed, favoring the richest with an OR of 1.62 (1.18-2.23). Having health insurance also remained in the final model, both in 2008 and 2015, proving to be an important marker for dental visits in the last year, with OR of 1.98 (1.47-2.67) and 1.69 (1.26-2.26), respectively

## DISCUSSION

The findings of this study reveal that, in general, the number of elderly people who visited a dentist increased from 2003 to 2015. It is observed that, despite the inequalities, the use of dental services increased, including for the most vulnerable groups, such as older individuals, non-white individuals, those without health insurance, and those with low levels of education and income. This increase can be reinforced by data from the National Oral Health Survey (SB Brasil) which, in 2023, showed that the Southeast region of the country had the highest percentage (32.11%) of elderly people seen by a dentist in the last year^22^.

Among the factors associated with the use of dental services, sex remains in the final model only in 2008. Previous studies that evaluated the use of dental services by the elderly Brazilian population, including the population of the city of São Paulo, do not highlight sex as a determinant of these inequalities^13^ ^,^ ^16^. This observation could explain why sex did not remain an important determinant of the use of oral health services by older adults in 2003 and 2015.

Age group is another important marker of these inequalities^10^ ^,^ ^12^ ^,^ ^13^ remaining associated with the final model in 2008 and 2015. It was observed that younger older adults (up to 74 years old) were more likely to have visited a dentist in the last year. This association is related to the number of missing teeth and the degree of dependence of these individuals, which is lower among younger older adults compared to older adults. This can be corroborated by Tôrres et al.^23^ and Warren et al.^24^, who demonstrated that advanced age among older adults is a risk factor for tooth loss; in addition to Moreira et al.^25^, Ribeiro et al.^26^ and Tani et al.^27^, who pointed out that functional capacity decreases with advancing age in samples of older people, including those from the city of São Paulo. However, it is known that the absence of dental elements alone does not justify less use or need for care by dental surgeons, nor does the degree of dependence, which, in fact, makes access to dental consultations difficult^28^ ^,^ ^29^ ^,^ ^30^.

Regarding race/skin color, although the literature points to important inequities regarding the oral health of the black population^12^ ^,^ ^31^, the present study identified an important association in favor of white individuals only for the year 2003. A possible explanation for why this inequality remained in the final model only in 2003 may be in relation to the possession of health insurance, collected only in 2008 and 2015, since the inclusion of this variable seems to have adjusted the model to the point of eliminating the isolated importance of the issue of race/skin color in relation to the use of dental services. However, it is worth noting that the inequalities regarding health insurance and education (which also remained in the final model in 2008 and 2015) are related to race/skin color inequalities in Brazill^32^ ^,^ ^33^.

The population with health insurance was more likely to have had a dental appointment in the last year. São Paulo is one of the cities with the highest coverage of beneficiaries of private health plans and agreements in Brazil, 48%, well above the national average (23%)^34^. As already mentioned, having a health insurance plan is an important marker of health inequalities. The association between having health insurance and greater use of dental services is evident in that the dental insurance or agreement itself encourages the individual to seek out the dentist more often because they do not have to pay for each appointment^35^. Furthermore, it is known that individuals with better socioeconomic status have greater access to supplementary health care, while groups with worse socioeconomic status remain exclusively dependent on the SUS. To reduce these inequalities, public policies, such as “Smiling Brazil”, should seek strategies to expand access to dental services at all levels of care, especially among those older^10^.

In this context, income is also an important marker of social inequalities. Thus, it was observed that in 2008, individuals with higher incomes were approximately 2.05 (1.02-4.10) times more likely to have visited a dentist in the last year compared to those with lower incomes, which seems to corroborate a significant inequality in the use of dental services, evidenced in other studies^10^ ^,^ ^12^. In 2015, this odds ratio reduced by 43%, suggesting a possible decrease in this inequality, which contributes to the reduction of negative outcomes such as tooth loss, dental pain and even infectious processes that affect the quality of life of older people^36^.

The findings of this study reveal that education is the main determinant for the use of dental services in the last year. Several studies show that individuals who have more years of education tend to have more information about health care and also a greater understanding of the importance of prevention, being more likely to use health services frequently beyond emergency situations. On the other hand, elderly individuals with less years of education, in general, tend to have less knowledge about the importance of preventive care related to oral health, leading to a late search for these services - which tends to occur only in more specific situations^1^ ^,^ ^37^ ^,^ ^38^ ^,^ ^39^.

The ISA-Capital data showed that this logic has lost strength. Across the three surveys studied, there was a reduction in the number of individuals with higher education who reported having used oral health services. Even so, the older population with higher education continued to be the one that used these services the most. This may be due to several factors, including the influence of socioeconomic status in this equation. Typically, older people with more years of education are also those with higher disposable income. As such, these people may have greater access to private consultations and, due to their healthier lifestyles, use more robust preventive methods, with more committed behaviors in relation to oral health, thus reducing the need to seek these services^40^ ^,^ ^41^.

Still regarding education, it is understood that elderly people with less time of education, in general, are also those with lower disposable income, which can influence the search for dental appointments in the last year. Furthermore, this population stratum tends to be concentrated in places with low coverage of the public health system, which makes it difficult to use dental services (both due to waiting time for an appointment and installed capacity), even when there is demand^42^.

As for the behavioral determinants observed in the 2015 survey, the frequency of toothbrushing and toothbrush replacement were shown to be factors associated with dental appointments only in the crude analyses. The adjustment by multiple analysis reinforced how much social determinants stand out in this discussion, even given the importance of these prevention measures for the main oral diseases, such as caries and periodontal disease^43^ ^,^ ^44^ ^,^ ^45^, reflecting the importance of intersectionality in the discussion on the use of dental services.

The findings of this study reinforce the idea that the use of dental services is influenced by a series of determinants. Among these, education and health insurance were shown to be the main factors associated with the use of oral health services by the elderly population of the most populated city in Brazil. Although these associations have lost strength over the years investigated, their direction still affects the most vulnerable strata, contributing to oral health outcomes that impair quality of life and active aging^23^ ^,^ ^24^ ^,^ ^36^.

This entire discussion presents some limitations that, although they do not invalidate the findings presented, should be considered. The ISA-Capital data collection instrument is a questionnaire in which the responses are self-reported. Although the care taken in selecting individuals, preparing the questionnaire and training the field team have minimized biases in selection, memory, interviewee and interviewer biases, these should not be completely discarded. Furthermore, the data presented refer to the year 2015, which is currently the most recent consolidated and available database of ISA-Capital.

It is necessary to recognize the limits of a health survey such as ISA-Capital, whose data collection, in addition to being quantitative and extensive, was not designed for the elderly population in particular. It is understood that unique aspects of each stratum investigated, as well as experiences with the health system, also permeate the issue of the use of oral health services. Therefore, qualitative approaches or mixed-method studies could enrich this entire discussion, based on the study of other variables and/or dimensions related to the use of these services, which are outside the methodological proposal of this study. Even so, the importance of ISA-Capital is highlighted as a population-based health survey, containing data that go beyond the SUS Health Information Systems, enabling discussions and new paths for health care.

Therefore, when evaluating the use of oral health services by the elderly population of the most populous municipality in Brazil and which reflects the social disparities throughout the country, it was observed that between 2003 and 2015 the factors associated with dental consultations, in general, lost strength over the period. However, the coverage of these services is still low and favors elderly people who are more educated; with good oral health behaviors; younger age, higher income and who have health insurance, revealing critical issues that deserve attention from managers, based on intersectoral actions that contribute to active and healthy aging. Furthermore, these findings reinforce the importance of the National Oral Health Policy, which ceased to be a health program in 2023 and officially became part of the list of SUS policies, with goals aimed at greater coverage of oral health services at all levels of care^11^.
