# Supplementary material for: Factors associated with the use of dental services by older people in the city of São Paulo, Brazil
Source: Rev Bras Epidemiol. 2025 Aug 4;28:e250042. doi: 10.1590/1980-549720250042.2 (PMC12321153; doi:10.1590/1980-549720250042.2)
Supplement: Translation

# Translation: Fatores associados ao uso dos serviços odontológicos pelas pessoas idosas do município de São Paulo, Brasil

**DOI:** 10.1590/1980-549720250042

**Authors:** Yan Nogueira Leite de Freitas, Cristian Arnecke Schröder, Camila Nascimento Monteiro, Edigê Felipe de Sousa Santos, Moisés Goldbaum, Chester Luiz Galvão Cesar, Marília Cristina Prado Louvison

**Affiliations:** I Universidade Federal do Amazonas, Faculdade de Odontologia - Manaus (AM), Brasil.; II Universidade de São Paulo, Faculdade de Saúde Pública - São Paulo (SP), Brasil.; III Hospital Sírio-Libanês - São Paulo (SP), Brasil.; IV Universidade de São Paulo, Faculdade de Medicina, Departamento de Medicina Preventiva - São Paulo (SP), Brasil.

**Keywords:** Idoso, Equidade no acesso aos serviços de saúde, Inquéritos epidemiológicos, Serviços de saúde bucal

## Abstract

**Objetivo:**

O estudo em questão teve como objetivo investigar os fatores associados ao uso dos serviços odontológicos pelas pessoas idosas residentes no município de São Paulo, Brasil.

**Métodos:**

Para tanto, foi conduzido um estudo transversal com análise de dados secundários provenientes das subamostras de pessoas idosas dos Inquéritos de Saúde no Município de São Paulo/Brasil (ISA-Capital), realizados em 2003, 2008 e 2015. Diversas variáveis foram testadas como fatores associados ao desfecho “consulta odontológica no último ano”. Razões de Chance ajustadas foram estimadas a partir de três modelos de regressão logística binária, um para cada ano.

**Resultados:**

O sexo se associou ao desfecho apenas em 2008, com Razão de Chance ajustada (RCa) de 0,65 (0,48-0,89) para os indivíduos do sexo feminino. Os indivíduos mais jovens (até 74 anos) tiveram mais chance de terem se consultado com o dentista em 2008 e 2015. A variável anos de estudo permaneceu associada ao desfecho nos três anos, revelando uma Razão de Chance ajustada de 8,22 (4,23-15,99) para os indivíduos com 12 anos ou mais de estudo em 2003. Essa medida diminuiu para 1,77 (1,25-2,51) em 2015. Cor/raça se manteve como fator associado somente em 2003 [RCa=1,50 (1,04-2,17)], a favor dos indivíduos autodeclarados brancos. Por outro lado, renda mensal e plano de saúde se mostraram fatores associados ao desfecho somente em 2008 e 2015.

**Conclusão:**

Os achados desse estudo apontam nós críticos que merecem atenção por parte dos gestores, a partir de ações intersetoriais que contribuam para um envelhecimento ativo e saudável.

## INTRODUÇÃO

Diversos estudos têm mostrado que as desigualdades no uso dos serviços de saúde representam um desafio global, mesmo em países que contam com sistemas de saúde de caráter universal, como é o caso do Brasil^1^ ^,^ ^2^ ^,^ ^3^ ^,^ ^4^ ^,^ ^5^ ^,^ ^6^. No que diz respeito aos serviços de saúde bucal, essas desigualdades são ainda mais marcantes, já que a odontologia, durante anos, esteve à margem das políticas públicas de saúde^7^ ^,^ ^8^. Sabe-se que a atenção à saúde bucal da população brasileira, por exemplo, nasceu a partir de uma lógica curativa e mutiladora - com foco em doenças e agravos em crianças e adolescentes -, refletindo um modelo de exclusão do acesso aos serviços odontológicos pelas pessoas idosas, com pouco impacto sobre os indicadores epidemiológicos^9^ ^,^ ^10^.

O Sistema Único de Saúde (SUS) brasileiro, desde 2004, tem traçado diretrizes a fim de superar as desigualdades no uso e acesso dos serviços odontológicos, que culminou na Política Nacional de Saúde Bucal (PNSB), também conhecida como “Brasil Sorridente”. O principal objetivo dessa política é diminuir as desigualdades na assistência à saúde bucal, ampliando o acesso da população aos serviços odontológicos de forma integral e com foco na Atenção Primária à Saúde (APS)^11^.

A partir da implementação da PNSB tem se observado um aumento do número de profissionais de saúde bucal atuantes no SUS, sobretudo com os incentivos destinados às Equipes de Saúde Bucal (eSB) na APS. Assim, observa-se, por exemplo, que em 2024 havia 35.827 eSB vinculadas ao SUS, garantindo 50,65% de cobertura da população brasileira. No município de São Paulo, para o mesmo ano, no mês de abril, havia 640 eSB contratadas, o que garantia apenas 18,56% de cobertura, de acordo com os dados disponibilizados pela plataforma e-Gestor AB do Ministério da Saúde (MS) (https://egestorab.saude.gov.br/). Observa-se, portanto, que apesar dos incentivos destinados à melhoria do acesso aos serviços odontológicos pelo MS nos últimos anos, a cobertura pela APS ainda é baixa na cidade de São Paulo^10^.

Nesse contexto, o uso dos serviços odontológicos têm sido objeto de investigação nas últimas décadas. Observa-se que indivíduos idosos, de menor renda e escolaridade, são os que têm maiores dificuldades para ir a uma consulta odontológica ou receber acompanhamento regular de um cirurgião-dentista^1^ ^,^ ^10^ ^,^ ^12^ ^,^ ^13^ ^,^ ^14^.

No caso do Brasil, a sua extensão territorial e as heterogeneidades regionais contribuem para a baixa cobertura dos serviços odontológicos oferecidos pelo SUS, principalmente dos serviços especializados, cuja demanda é alta pela população idosa. Nesse contexto, essas desigualdades podem ser percebidas ainda mais facilmente, uma vez que boa parte da demanda é direcionada à saúde suplementar ou às consultas particulares por pagamento direto, reforçando as disparidades no acesso à saúde bucal nesse estrato populacional. Logo, entende-se que essas desigualdades são melhor explicadas pelos determinantes socioeconômicos e demográficos do que pela saúde bucal desses indivíduos^13^.

Dentre as diferentes realidades do Brasil, o município de São Paulo se destaca por apresentar o maior número absoluto de pessoas idosas do país^15^. Quanto às desigualdades no uso dos serviços odontológicos nesse município, Monteiro et al.^16^ observaram um aumento do uso de serviços de saúde bucal pela população adulta entre 2003 e 2008. No entanto, esse aumento esteve associado aos indivíduos de cor branca, maior escolaridade, maior renda, melhores condições de moradia e posse de plano de saúde. Os autores ainda destacam que houve um pequeno aumento do uso desses serviços no setor público pela população de menor condição socioeconômica. Portanto, verificar se esses e outros determinantes também se relacionam ao uso desses serviços por um grupo etário que carece de atenção à saúde bucal^1^ ^,^ ^12^ ^,^ ^13^, no contexto da cidade de São Paulo, pode contribuir para a discussão de ações e/ou políticas a fim de melhorar este panorama.

Diante desse cenário, este estudo teve como objetivo investigar os fatores associados ao uso dos serviços odontológicos pelas pessoas idosas do município de São Paulo, Brasil, nos anos de 2003, 2008 e 2015.

## MÉTODOS

Para responder ao objetivo proposto, foi conduzido um estudo transversal seriado com análise de dados secundários provenientes das subamostras de pessoas idosas dos Inquéritos de Saúde no Município de São Paulo/Brasil (ISA-Capital), realizados em 2003, 2008 e 2015.

De acordo com o Instituto Brasileiro de Geografia e Estatística (IBGE), em 2022 a população do município de São Paulo era de 11.451.999 habitantes, dos quais 2.027.003 eram pessoas idosas, com 60 anos ou mais de idade, representando 17,7% da população residente^15^.

O ISA-Capital é um estudo transversal de base populacional realizado no município de São Paulo, por meio de entrevistas domiciliares. Em 2003 foram entrevistados 3.357 indivíduos, dos quais 872 (25,97%) pertenciam à faixa etária dos 60 anos ou mais, a partir de uma amostragem estratificada por conglomerados em dois estágios (os setores censitários e os domicílios)^17^. Seguindo essa metodologia, em 2008 uma amostragem estratificada pelos mesmos conglomerados selecionou 3.271 indivíduos, dos quais 924 (28,25%) eram pessoas idosas^18^. No ano de 2015, a amostragem manteve a lógica de conglomerados por dois estágios, mas pela primeira vez os setores censitários foram agrupados de acordo com as cinco Coordenadorias Regionais de Saúde do município: Norte, Sul, Leste, Centro-Oeste e Sudeste. Dessa forma, foram selecionados 3.184 indivíduos para compor a amostra, cuja faixa etária idosa correspondeu a 32% desses, totalizando 1.019 indivíduos^19^. Maiores detalhes metodológicos desses inquéritos podem ser encontrados no site do ISA-Capital (https://www.fsp.usp.br/isa-capital/metodologia/).

Para a tabulação de dados foram considerados os questionários aplicados em 2003, 2008 e 2015, organizados em blocos temáticos compostos, em sua maioria, por questões de múltipla escolha. Os dados foram coletados por entrevistadores treinados e as perguntas, respondidas diretamente pelos moradores dos domicílios sorteados.

Como variável desfecho foi considerada a pergunta referente à consulta odontológica no último ano, categorizada em sim/não, que foi retirada do bloco G dos questionários, que diz respeito ao uso dos serviços de saúde. As variáveis independentes foram selecionadas conforme o Modelo Comportamental de Andersen^20^ e, também, de acordo com a literatura, por serem reconhecidas como fatores que se associam ao uso dos serviços odontológicos^21^. Portanto, foram consideradas como independentes: sexo (masculino/feminino); idade (até 74 anos/75 anos ou mais); cor/raça (branca/preta/parda/amarela/indígena); situação conjugal (casado(a)/não casado(a)); anos de estudo (nunca ou até 3 anos/4 a 7 anos/8 a 11 anos/12 ou mais anos); renda mensal (menos de um 1 salário mínimo (SM)/entre 1 e 2,5 SM/mais de 2,5 até 6 SM/mais de 6 SM); problema de saúde nos últimos 15 dias (sim/não) e multimorbidade (sim/não), considerada como duas ou mais doenças crônicas autorrelatadas.

Destaca-se que algumas variáveis independentes não foram consideradas para os três inquéritos, pois houve mudanças ao longo dos anos nos questionários. Assim, a autopercepção da saúde geral (positiva/negativa) foi considerada para os inquéritos de 2003 e 2015; por outro lado, a autopercepção da saúde bucal somente para o inquérito de 2015. A posse de plano de saúde (sim/não) foi considerada, apenas, para os inquéritos de 2008 e 2015. A polifarmácia (sim/não), considerada como o uso concomitante de quatro ou mais medicamentos diariamente, foi selecionada para os inquéritos de 2003 e 2015. Já a frequência da escovação dentária (nunca/não escova todos os dias/1 vez ao dia/2 ou mais vezes ao dia) e frequência com que troca a escova de dente (menos de 3 meses/entre 3 e menos de 6 meses/entre 6 meses e menos de 1 ano/mais de 1 ano/nunca) foram variáveis observadas somente no inquérito de 2015.

Inicialmente, foi conduzida uma análise descritiva para cada uma das três subamostras investigadas, de onde se observou as prevalências do desfecho entre as categorias das variáveis independentes, cujas significâncias foram testadas a partir do Teste X^2^ de Pearson, para um nível de 95% de confiança. A seguir, foram testadas as medidas de efeito (Razões de Chance-RC) brutas e ajustadas, considerando o mesmo nível de confiança. As medidas ajustadas foram estimadas a partir de uma regressão logística binária, mantendo o nível de confiança. Para compor a análise múltipla, foram selecionadas as variáveis com p≤0,25 na análise bruta.

### Declaração de disponibilidade de dados

O conjunto de dados que dá suporte aos resultados deste estudo não está disponível publicamente.

## RESULTADOS

O uso dos serviços odontológicos se distribuiu de forma heterogênea ao longo dos três inquéritos. As variáveis cor/raça, anos de estudo e renda estiveram associadas ao desfecho nos três anos estudados (com p-valores variando de ≤0,001 a 0,01), onde a prevalência de consultas odontológicas no último ano foi maior entre os indivíduos autodeclarados brancos e amarelos, com 12 ou mais anos de estudo e que recebiam mais de 6 SM ao mês. A posse de plano de saúde também se associou ao desfecho em 2008 e 2015 (p≤0,001), onde indivíduos que relataram possuir plano de saúde se consultaram mais com o dentista, conforme Tabela 1.

**Tabela 1.** Análise descritiva dos dados referentes ao ISA-Capital 2003, 2008 e 2015. São Paulo, Brasil, 2025.

Variáveis	Consulta odontológica último ano (2003) n=872			Consulta odontológica último ano (2008) n=924			Consulta odontológica último ano (2015) n=1.019		
	Sim (%)	Não (%)	p-valor*	Sim (%)	Não (%)	p-valor*	Sim (%)	Não (%)	p-valor*
Sexo									
Masculino	122 (29,8)	288 (70,2)	0,381	128 (36,0)	228 (64,0)	0,153	147 (38,0)	240 (62,0)	0,066
Feminino	119 (27,0)	321(73,0)		231 (40,7)	337 (59,3)		277 (43,8)	355 (56,2)	
Idade (anos)									
Até 74	189 (29,6)	450 (70,4)	0,168	259 (42,1)	356 (57,9)	**0,004**	339 (44,7)	419 (55,3)	**0,001**
75 ou mais	52 (24,6)	159 (75,4)		100 (32,4)	209 (67,6)		85 (32,6)	176 (67,4)	
Cor/Raça									
Branca	188 (31,9)	401 (68,1)	**0,01**	277 (42,3)	378 (57,7)	**0,0002**	269 (45,1)	328 (54,9)	**0,001**
Preta	11(18,6)	48(81,4)		14 (26,4)	39 (73,6)		24 (30,4)	55 (69,6)	
Parda	35 (20,1)	139 (79,9)		53 (28,6)	132 (71,4)		77 (34,2)	148 (65,8)	
Amarela	7 (28,0)	18 (72,0)		13 (46,4)	15 (53,6)		21 (61,8)	13 (38,2)	
Anos de estudo (anos)									
Nunca/até 3	64 (17,9)	294 (82,1)	**≤0,001**	79 (25,2)	235 (74,8)	**≤0,001**	49 (25,0)	147 (75,0)	**≤0,001**
4 a 7	88 (28,6)	220 (71,4)		124 (35,5)	225 (64,5)		120 (37,6)	199 (62,4)	
8 a 11	44 (38,9)	69 (61,1)		103 (54,8)	85 (45,2)		111 (43,0)	147 (57,0)	
12 ou mais	42 (73,7)	15 (26,3)		52 (73,2)	19 (26,8)		144 (58,5)	102 (41,5)	
Renda mensal^†^ (salário mínimo)									
Menos de 1	25 (25,0)	75 (75,0)	**≤0,001**	13 (22,4)	45 (77,6)	**≤0,001**	41 (31,8)	88 (68,2)	**≤0,001**
Entre 1 e 2,5	44 (18,3)	196 (81,7)		105 (31,0)	234 (69,0)		97 (33,0)	197 (67,0)	
Mais de 2,5 a 6	91 (31,1)	202 (68,9)		80 (44,2)	101 (55,8)		108 (42,4)	147 (57,6)	
Mais de 6	81 (37,3)	136 (62,7)		161 (46,5)	185 (53,5)		178 (52,2)	163 (47,8)	
Plano de saúde									
Sim	-	-		223 (50,3)	220 (49,7)	**≤0,001**	306 (48,8)	321 (51,2)	**≤0,001**
Não	-	-		136 (28,3)	345 (71,7)		118 (30,3)	271 (69,7)	

*significância do X^2^ de Pearson; ^†^salário-mínimo em 2003 era R$ 240; em 2008 era R$ 415; e em 2015, R$ 788.

Os números em negrito destacam associações significativas do ponto de vista estatístico.

Quanto às medidas de efeito, no geral, observam-se Razões de Chance (RC) elevadas e que diminuem ao longo do tempo. A variável anos de estudo exemplifica esses achados, pois indivíduos que em 2003 haviam estudado por 12 ou mais anos tinham cerca de 8,36 (4,54-15,40) vezes mais chance de ter se consultado com o dentista no último ano, se comparados aos que nunca estudaram ou estudaram por apenas três anos. Quando ajustada para as demais variáveis, essa estatística cai para 8,22 (4,23-15,99). No entanto, em 2015 observa-se uma diminuição dessas medidas, com RC bruta de 2,49 (1,85-3,33) e a ajustada no valor de 1,77 (1,25-2,51), indicando uma possível redução das desigualdades no uso dos serviços de saúde bucal em relação à escolaridade nas subamostras estudadas, conforme Tabelas 2 e 3.

**Tabela 2.** Razões de Chance brutas do ISA-Capital 2003, 2008 e 2015. São Paulo, Brasil, 2025.

Variáveis	Consulta odontológica último ano (2003) n=872			Consulta odontológica último ano (2008) n=924			Consulta odontológica último ano (2015) n=1.019		
	RCb	(IC95%)	p-valor*	RCb	(IC95%)	p-valor*	RCb	(IC95%)	p-valor*
Sexo									
Masculino	1,14	(0,85-1,54)	0,381	0,82	(0,62-1,08)	0,153	0,78	(0,61-1,02)	0,066
Feminino	1			1			1		
Idade (anos)									
Até 74	1,28	(0,90-1,83)	0,168	**1,52**	**(1,14-202)**	0,004	**1,67**	**(1,25-2,25)**	0,001
75 ou mais	1			1			1		
Cor/Raça									
Branca	**1,84**	**(1,30-2,60)**	0,001	**1,67**	**(1,23-2,26)**	0,001	**1,41**	**(1,09-1,82)**	0,008
Parda/Preta	1			1			1		
Situação conjugal									
Casado	1,21	(0,87-1,67)	0,229	1,00	(0,77-1,31)	0,957	1,02	(0,79-1,30)	0,894
Não casado	1			1			1		
Anos de estudo (anos)									
Nunca/até 3	1			1			1		
4 a 7	1,02	(0,75-1,39)	0,915	0,79	(0,61-1,05)	0,106	0,79	(0,60-1,03)	0,081
8 a 11	**1,75**	**(1,16-2,64)**	0,007	**2,27**	**(1,64-3,14)**	≤0,001	1,08	(0,81-1,44)	0,594
12 ou mais	**8,36**	**(4,54-15,40)**	≤0,001	**4,87**	**(2,83-8,38)**	≤0,001	**2,49**	**(1,85-3,33)**	≤0,001
Renda mensal^†^ (salário mínimo)									
Menos de 1	1			1			1		
Entre 1 e 2,5	**0,42**	**(0,33-0,68)**	≤0,001	**0,58**	**(0,44-0,78)**	≤ 0,001	**0,60**	**(0,45-0,80)**	≤0,001
Mais de 2,5 a 6	1,22	(0,90-1,67)	0,204	1,32	(0,95-1,83)	0,1	1,04	(0,78-1,39)	0,781
Mais de 6 S	**1,76**	**(1,27-2,44)**	0,001	**1,67**	**(1,27-2,19)**	≤ 0,001	**1,92**	**(1,47-2,50)**	≤0,001
Problema de saúde nos últimos 15 dias									
Sim	0,84	(0,60-1,19)	0,332	0,91	(0,67-1,24)	0,559	1,03	(0,77-1,38)	0,853
Não	1			1			1		
Autopercepção saúde geral									
Positiva	1,33	(0,86-2,03)	0,196	-	-		**1,38**	**(1,07-1,78)**	0,014
Negativa	1			-	-		1		
Multimorbidade									
Sim	0,99	(0,74-1,34)	0,992	0,84	(0,64-1,11)	0,222	0,89	(0,69-1,15)	0,380
Não	1			1			1		
Polifarmácia									
Sim	0,84	(0,57-1,24)	0,373	-	-		1,15	(0,89-1,48)	0,278
Não	1			-	-		1		
Plano de saúde									
Sim	-	-		**2,57**	**(1,96-3,38)**	≤ 0,001	**2,19**	**(1,68-2,86)**	≤0,001
Não	-	-		1			1		
Autopercepção saúde bucal									
Positiva	-	-		-	-		**1,33**	**(1,03-1,73)**	0,03
Negativa	-	-		-	-		1		
Frequência de escovação dentária									
Não escova	-	-		-	-		1		
1 vez ao dia	-	-		-	-		**0,32**	**(0,21-0,51)**	≤0,001
2 ou + vezes ao dia	-	-		-	-		**3,37**	**(2,22-5,11)**	≤0,001
Substituição da escova dentária (meses)									
Menos de 3	-	-		-	-		1		
Entre 3 e 6	-	-		-	-		1,27	(0,98-1,63)	0,07
6 ou mais	-	-		-	-		**0,57**	**(0,42-0,78)**	≤0,001

RCb: Razões de Chance brutas.

*significância da Razão de Chance Bruta; ^†^salário-mínimo em 2003 era R$ 240; em 2008 era R$ 415; e em 2015, R$ 788.

Os números em negrito apontam Razões de Chance significativas de acordo com o intervalo de confiança.

**Tabela 3.** Razões de Chance ajustadas do ISA-Capital 2003, 2008 e 2015. São Paulo, Brasil, 2025.

Variáveis	Consulta odontológica último ano (2003) n=872			Consulta odontológica último ano (2008) n=924			Consulta odontológica último ano (2015) n=1.019		
	RCa	(IC95%)	p-valor*	RCa	(IC95%)	p-valor*	RCa	(IC95%)	p-valor*
Sexo									
Masculino	-	-		**0,65**	**(0,48-0,89)**	0,008	0,813	(0,61-1,08)	0,148
Feminino	-	-		1	-		1	-	
Idade (anos)									
Até 74	1,13	(0,78-1,65)	0,51	**1,50**	**(1,09-2,04)**	0,012	**1,60**	**(1,17-2,20)**	0,003
75 ou mais	1	-		1	-		1		
Cor/Raça									
Branca	**1,50**	**(1,04-2,17)**	0,032	1,16	(0,83-1,61)	0,38	1,23	(0,93-1,61)	0,143
Parda/Preta	1	-		1	-		1	-	
Situação conjugal									
Casado	0,99	(0,70-1,39)	0,935						
Não casado	1	-							
Anos de estudo (anos)									
Nunca/até 3	1	-		1	-		1		
4 a 7	-	-		1,53	(1,08-2,16)	0,018	1,12	(0,82-1,53)	0,487
8 a 11	**1,94**	**(1,25-3,00)**	0,003	**2,83**	**(1,86-4,31)**	≤0,001	-	-	
12 ou mais	**8,22**	**(4,23-15,99)**	≤0,001	**5,93**	**(3,15-11,17)**	≤0,001	**1,77**	**(1,25-2,51)**	0,001
Renda mensal^†^ (salário mínimo)									
Menos de 1	1	-		1	-		1		
Entre 1 e 2,5	0,79	0,45-1,41)	0,433	1,64	(0,82-3,30)	0,163	0,99	(0,71-1,40)	0,982
Mais de 2,5 a 6	1,41	(0,82-2,41)	0,213	1,95	(0,93-4,01)	0,077	-	-	
Mais de 6	1,11	(0,62-1,98)	0,724	**2,05**	**(1,02-4,10)**	0,043	**1,62**	**(1,18-2,23)**	0,003
Autopercepção saúde geral									
Positiva	1,00	(0,64-1,57)	0,99				1,11	(0,84-1,48)	0,465
Negativa	1	-					1	-	
Multimorbidade									
Sim	-	-		1,02	(0,75-1,39)	0,895			
Não	-	-		1	-				
Plano de saúde									
Sim	-	-		**1,98**	**(1,47-2,67)**	≤ 0,001	**1,69**	**(1,26-2,26)**	≤0,001
Não	-	-		1	-		1	-	
Autopercepção saúde bucal									
Positiva							1,14	(0,86-1,52)	0,351
Negativa							1	-	
Frequência de escovação dentária									
Não escova	-						1	-	
1 vez ao dia	-	-		-	-		1,11	(0,34-3,62)	0,858
2 ou + vezes ao dia	-	-		-	-		2,51	(0,824-7,63)	0,105
Substituição da escova dentária (meses)									
Menos de 3	-						1	-	
Entre 3 e 6	-	-		-			1,12	(0,83-1,51)	0,471
6 ou mais	-						0,71	(0,48-1,04)	0,076

*significância da Razão de Chance ajustada; ^†^salário-mínimo em 2003 era R$ 240; em 2008, era R$ 415; e em 2015, R$ 788.

Os números em negrito apontam Razões de Chance significativas de acordo com o intervalo de confiança.

A Tabela 3 apresenta o modelo de melhor ajuste na análise múltipla para cada um dos inquéritos estudados. Assim, observa-se, por exemplo, que em 2003 os indivíduos que se autodeclararam brancos tinham mais chances de terem se consultado com dentista no último ano, se comparados aos indivíduos pardos ou pretos [RC=1,50 (1,04-2,17)]. Já em relação ao sexo, em 2008 os indivíduos do sexo masculino tinham menos chances de terem se consultado com o dentista no último ano [RC=0,65 (0,48-0,89)]. A renda se manteve associada ao desfecho nos dois últimos inquéritos, revelando que em 2008 os indivíduos que recebiam mais de 6 SM tinham cerca de 2,05 (1,09-2,11) vezes mais chance de ter se consultado com o dentista no último ano se comparados ao extrato de menor renda. Em 2015 observa-se uma ligeira queda dessa possível desigualdade por renda, favorecendo os mais ricos com RC de 1,62 (1,18-2,23). A posse de plano de saúde também permaneceu no modelo final, tanto em 2008 como em 2015, se mostrando um importante marcador para a consulta odontológica no último ano, com RC de 1,98 (1,47-2,67) e 1,69 (1,26-2,26), respectivamente.

## DISCUSSÃO

Os achados do presente estudo revelam que, em geral, de 2003 a 2015 o número de pessoas idosas que se consultaram com o dentista aumentou. Observa-se que, apesar das desigualdades, o uso dos serviços odontológicos aumentou, inclusive para os grupos mais vulneráveis, como os indivíduos mais velhos, não brancos, sem plano de saúde e de baixa escolaridade e renda. Esse aumento pode ser reforçado pelos dados da Pesquisa Nacional de Saúde Bucal (SB Brasil) que, em 2023, mostrou que a região Sudeste do país apresentou o maior percentual (32,11%) de pessoas idosas atendidas por um dentista no último ano^22^.

Entre os fatores associados ao uso dos serviços odontológicos, o sexo permanece no modelo final apenas no ano de 2008. Estudos anteriores que avaliaram o uso dos serviços odontológicos pela população idosa brasileira, inclusive da população do município de São Paulo, não destacam o sexo como determinante dessas desigualdades^13^ ^,^ ^16^. Essa observação poderia justificar o porquê de o sexo não ter permanecido como um determinante importante do uso dos serviços de saúde bucal pelas pessoas idosas em 2003 e 2015.

A faixa etária é outro marcador importante dessas desigualdades^10^ ^,^ ^12^ ^,^ ^13^, mantendo-se associada ao modelo final em 2008 e 2015. Observou-se que as pessoas idosas mais jovens (até os 74 anos) contabilizavam mais chances de terem se consultado com o dentista no último ano. Tal associação guarda relação com o número de dentes perdidos e o grau de dependência desses indivíduos, que é menor entre os idosos mais jovens se comparado aos idosos longevos. Isso pode ser corroborado por Tôrres et al.^23^ e Warren et al.^24^, que demonstraram que a idade avançada entre pessoas idosas é fator de risco para a perda dentária; além de Moreira et al.^25^, Ribeiro et al.^26^ e Tani et al.^27^, que apontaram que a capacidade funcional diminui com o avançar da idade em amostras de pessoas idosas, inclusive do município de São Paulo. No entanto, sabe-se que a ausência de elementos dentários por si só não justifica menor uso ou necessidade de cuidados por parte dos cirurgiões dentistas, tampouco o grau de dependência, que, na verdade, dificulta o acesso a consultas odontológicas^28^ ^,^ ^29^ ^,^ ^30^.

Em relação à raça/cor da pele, apesar de a literatura apontar importantes iniquidades no que diz respeito à saúde bucal da população negra^12^ ^,^ ^31^, o presente estudo identificou uma associação importante a favor dos indivíduos brancos somente para ano de 2003. Uma possível explicação para que essa desigualdade tenha permanecido no modelo final somente em 2003 pode ser em relação à posse de plano de saúde, coletada apenas em 2008 e 2015, uma vez que a inclusão dessa variável parece ter ajustado o modelo a ponto de eliminar a importância isolada da questão raça/cor frente ao uso dos serviços odontológicos. No entanto, cabe destacar que as desigualdades referentes a plano de saúde e escolaridade (que também se manteve no modelo final em 2008 e 2015) se relacionam às desigualdades de raça/cor no Brasil^32^ ^,^ ^33^.

A população com plano de saúde apresentou mais chance de ter ido uma consulta odontológica no último ano. São Paulo é uma das cidades com maior cobertura de beneficiários de planos e convênios de saúde privados no Brasil, 48%, bem acima da média nacional (23%)^34^. Como já mencionado, a posse de plano de saúde é um importante marcador de desigualdades em saúde. A associação entre a posse de plano de saúde e o maior uso dos serviços odontológicos evidencia-se na medida em que o plano ou convênio odontológico por si só estimula o indivíduo a procurar mais o dentista por não ter que pagar por cada consulta^35^. Ademais, sabe-se que indivíduos com melhor condição socioeconômica possuem maior acesso a cuidados de saúde suplementar, enquanto grupos com pior condição socioeconômica permanecem dependentes exclusivamente do SUS. Para reduzir essas desigualdades, políticas públicas, como o “Brasil Sorridente”, devem buscar estratégias para ampliar o acesso a serviços odontológicos em todos os níveis de atenção, principalmente entre as pessoas idosas^10^.

Nesse contexto, a renda também é um importante marcador de desigualdades sociais. Assim, observou-se que em 2008 os indivíduos de maior renda tinham cerca de 2,05 (1,02-4,10) vezes mais chance de ter se consultado com o dentista no último ano se comparados ao extrato de menor renda, o que parece corroborar com uma importante desigualdade no uso de serviços odontológicos, evidenciada em outros estudos^10^ ^,^ ^12^. Em 2015 essa razão de chances reduz em 43%, sugerindo uma possível diminuição dessa desigualdade, o que contribui para a redução de desfechos negativos como a perda dentária, dor dentária e até processos infecciosos que afetam a qualidade de vida das pessoas idosas^36^.

Os achados do presente estudo revelam a escolaridade como o principal determinante para o uso dos serviços odontológicos no último ano. Estudos diversos mostram que indivíduos que possuem maior tempo de estudo tendem a ter mais informações acerca dos cuidados com a saúde e, também, maior compreensão sobre a importância da prevenção, sendo mais propensos a utilizar frequentemente os serviços de saúde para além das situações de urgência. Por outro lado, pessoas idosas com menor tempo de estudo, em geral, tendem a apresentar menos conhecimento sobre a importância dos cuidados preventivos relacionados à saúde bucal, levando a uma busca tardia por esses serviços - o que tende a ocorrer apenas em situações mais específicas^1^ ^,^ ^37^ ^,^ ^38^ ^,^ ^39^.

Os dados do ISA-Capital mostraram que essa lógica tem perdido força. Ao longo dos três inquéritos estudados, observa-se uma redução no número de indivíduos de maior escolaridade que relataram ter usado os serviços de saúde bucal. Mesmo assim, a população idosa com maior escolaridade continuava sendo a que mais utilizava esses serviços. Isso pode acontecer por diversos fatores, inclusive devido à influência da posição socioeconômica nesta equação. Normalmente, pessoas idosas com mais anos de estudo também são aquelas que possuem maior renda disponível. Dessa maneira, essas pessoas podem ter maior acesso às consultas privadas e, devido aos seus estilos de vida mais saudáveis, utilizam de métodos preventivos mais robustos, com comportamentos mais comprometidos em relação à saúde bucal e diminuindo, assim, a necessidade de procura por esses serviços^40^ ^,^ ^41^.

Ainda em relação à escolaridade, entende-se que pessoas idosas com menor tempo de estudo, em geral, também são aquelas com menor renda disponível, o que pode influenciar na procura por consultas odontológicas no último ano. Ademais, esse estrato populacional tende a se concentrar em locais de baixa cobertura assistencial do sistema de saúde público, o que dificulta o uso dos serviços odontológicos (tanto por tempo de espera por uma consulta, quanto por capacidade instalada), mesmo quando há procura^42^.

Quanto aos determinantes comportamentais observados no inquérito de 2015, a frequência de escovação dentária e a substituição da escova dentária se mostraram fatores associados às consultas odontológicas somente nas análises brutas. O ajuste pela análise múltipla reforçou o quanto os determinantes sociais se destacam nessa discussão, mesmo diante da importância dessas medidas de prevenção para as principais doenças bucais, como a cárie e a doença periodontal^43^ ^,^ ^44^ ^,^ ^45^, refletindo a importância da interseccionalidade na discussão sobre o uso dos serviços odontológicos.

Os achados do presente estudo reforçam a ideia de que o uso dos serviços odontológicos perpassa por uma série de determinantes. Dentre esses, a escolaridade e o plano de saúde se mostraram os principais fatores associados ao uso dos serviços de saúde bucal pela população idosa do município mais populoso do Brasil. Apesar dessas associações terem perdido força ao longo dos anos investigados, a direção delas ainda afeta os estratos de maior vulnerabilidade, contribuindo para desfechos em saúde bucal que prejudicam a qualidade de vida e o envelhecimento ativo^23^ ^,^ ^24^ ^,^ ^36^.

Toda essa discussão apresenta algumas limitações que, apesar de não invalidar os achados apresentados, devem ser consideradas. O ISA-Capital tem como instrumento de coleta de dados um questionário em que as respostas são autorreferidas. Embora o cuidado com a seleção dos indivíduos, elaboração do questionário e o treinamento da equipe de campo tenham minimizado os vieses de seleção, memória, do entrevistado e do entrevistador, os mesmos não devem ser completamente descartados. Ademais, os dados apresentados se referem até o ano de 2015, que é, no momento, a mais recente base de dados consolidados e disponíveis do ISA-Capital.

É preciso reconhecer os limites de um inquérito de saúde como o ISA-Capital, cuja coleta de dados, além de quantitativa e extensa, não foi pensada para a população idosa em específico. Entende-se que aspectos singulares de cada estrato investigado, bem como experiências vivenciadas junto ao sistema de saúde, também permeiam a questão do uso dos serviços de saúde bucal. Portanto, abordagens qualitativas ou estudos de métodos mistos poderiam enriquecer toda essa discussão, a partir do estudo de outras variáveis e/ou dimensões relativas ao uso desses serviços, que estão fora da proposta metodológica do presente estudo. Ainda assim, destaca-se a importância do ISA-Capital enquanto inquérito de saúde de base populacional, contendo dados que vão além dos Sistemas de Informação em Saúde do SUS, possibilitando discussões e novos caminhos para atenção à saúde.

Portanto, ao avaliar o uso dos serviços de saúde bucal pela população idosa do município mais populoso do Brasil e que reflete as disparidades sociais ao redor do país, observou-se que entre 2003 e 2015 os fatores associados às consultas odontológicas, no geral, perderam força ao longo do período. No entanto, a cobertura desses serviços ainda é baixa e favorece as pessoas idosas mais escolarizadas; com bons comportamentos de saúde bucal; de menor idade, maior renda e que possuem planos de saúde, revelando nós críticos que merecem atenção por parte dos gestores, a partir de ações intersetoriais que contribuam para um envelhecimento ativo e saudável. Ademais, esses achados reforçam a importância da Política Nacional de Saúde Bucal, que deixou de ser um programa de saúde em 2023 e passou a compor oficialmente o rol de políticas do SUS, com metas que visam maior cobertura dos serviços de saúde bucal em todos os níveis de atenção^11^.
